# Editorial: Effective options regarding spay or neuter of dogs

**DOI:** 10.3389/fvets.2024.1442805

**Published:** 2024-06-18

**Authors:** Lynette A. Hart, Benjamin L. Hart, Michelle A. Kutzler, Kate N. Atema

**Affiliations:** ^1^Department of Population Health and Reproduction, School of Veterinary Medicine, University of California, Davis, Davis, CA, United States; ^2^Department of Anatomy, Physiology, and Cell Biology, School of Veterinary Medicine, University of California, Davis, Davis, CA, United States; ^3^Department of Animal and Rangeland Sciences, Oregon State University, Corvallis, OR, United States; ^4^International Companion Animal Management Coalition, Cambridge, United Kingdom

**Keywords:** behavior, cancer, castration, disease, ethics, health, joint disorder, welfare

## Introduction

Spaying of females and castration of males, collectively termed neutering, became a common practice for many dogs in the United States in recent decades, aimed at reducing relinquishment of dogs to animal shelters. Often performed at early ages, neutering of dogs prior to adoption from shelters often became a local or state legal requirement. However, scattered evidence began accumulating of some adverse effects of neutering with increases in certain cancers (1–5) and joint disorders (6). Emerging issues pertaining to spay-neuter provoked us to spearhead this Research Topic to provide an accessible resource to better understand current attitudes regarding neutering, surgical and alternative methods for neutering, and the behavioral and health effects of neutering dogs. These 16 research articles clarify some of the complexities associated with making decisions regarding spay and neuter of dogs; they have been of high interest to the general public.

## Demographics and attitudes regarding neutered dogs

The research literature relevant to canine spay-neuter is widely dispersed and publications can be challenging to locate. Focusing on studies assessing attitudes to spaying of female dogs, Fausak searched three databases, CAB Direct, PubMed, and Scopus, to identify and characterize journals publishing most of the articles on this topic. For the 84 out of 642 articles identified as relevant on attitudes to female canine spaying, six journals each had published at least four articles. These journals either represented veterinary organizations or focused on reproduction. Most of these articles presented perspectives of veterinarians and a few had surveyed pet owners.

Changing patterns of dog ownership in Costa Rica were investigated by Flockhart et al. in 2020, with a goal of comparing results with earlier studies. They found a high rate of dog ownership: 76% of households, a sharp increase from prior studies reporting 53%, 50%, and 49% of homes with dogs, in 2000, 2003, and 2011, respectively. People living on farms had the highest rate of ownership: 92%. Respondents reported that 67% of female dogs and 61% of males were neutered. This high neutering rate was a sharp increase from only 18% and 36% that previously had been reported in 2003 and 2011, respectively. Data provided evidence that “responsible pet owner” behavior had been increasing in Costa Rica.

Working in the Australian Capital Territory, Orr and Jones explored compliance with the legal requirement since 2001 for prepubertal desexing (neutering) for all cats and dogs. All cats are required to be desexed by 3 months of age, and all dogs by 6 months of age. Yet, very few of 52 responding veterinarians recommended that clients have their cat desexed at 3 months of age, as legally required. Almost half of veterinarians thought prepubertal desexing of cats was appropriate to prevent overpopulation. But over one-third of responding veterinarians did not realize that prepubertal desexing was mandatory, suggesting that the law may be poorly understood and supported by veterinarians.

Considering the possible impact of COVID-19 on elective neutering surgeries for dogs and cats, Guerios et al.  explored whether the disease onset of COVID-19 was associated with a reduction in these surgeries. Records from 212 clinics reported 190,818 fewer neuter surgeries during years 2021-2022 than would have been expected if 2019 neutering levels had been maintained. Total surgeries for dogs decreased 19% and 14% in 2020 and 2021, respectively; surgeries for cats decreased 10% and 3%. If a similar rate of decline were experienced by all spay/neuter providers in the US, there would have been an overall deficit of more than 2.7 million spay/neuter surgeries during that 2-year period.

Exploring the possible effect of widespread neutering on the breeding of dogs, Dawson et al. presented a rationale arguing that a fundamental shift is required with collaboration of breeders and owners to promote inclusion of “proven” companion dogs in the gene pool–those likely to become good companions—rather than selecting dogs to breed that meet conformation or working/sporting standards. Details on breeding choices and puppy rearing should be documented, and based on knowledge, with breeders tested for suitability. Then, available companion dogs would more successfully meet the needs of modern urban dog owners. A new model is proposed, whereby responsible owners and breeders work together to produce dogs that are most suited for life as human companions.

## Methods of altering reproductive capacity

Gonads are reproductive organs but also endocrine glands affecting normal metabolic, behavioral, and musculoskeletal health. Lifelong extremely heightened luteinizing hormone (LH) secreted after neutering, contributes to various disorders for both sexes (7). Thus, as a method to avoid the excessive LH secretion, Kutzler's review describes two gonad sparing surgeries that can be used to sterilize dogs. For females, ovary-sparing hysterectomy removes the uterus and much of the cervix, while leaving the ovaries intact. These females still experience regular estrous cycles but there is not vaginal discharge or risk for pyometra. For males, vasectomy is a quick surgery that involves bilateral removal and/or occlusion of a portion of the vas deferens. When performed on pediatric patients, these surgeries do not interfere with pubertal maturation. Once a dog is fully mature, the owner can still decide to remove the gonads.

Silva et al. investigated a simplified method of pain control with sedation and local anesthesia for use during castration of male dogs, avoiding general anesthesia as is done in humans and other species. Sedating dogs intramuscularly with xylazine and a sub-anesthetic dose of ketamine and administering lidocaine at the incision site and intratesticularly allowed dogs to be castrated humanely. This method avoided the expense of general anesthesia and the need for hospitalization.

Darounkolaei et al. compared ovariohysterectomy in large, deep-chested, mixed breed dogs performed in a conventional technique vs. an instrument shank-assisted ovariohysterectomy that was conducted by a single person. The new surgical method required use of four hemostats and two forceps and required less time to complete: averaging 34.7 min vs. 47.4 min. They concluded that the new method performed by just one person resulted in better time efficiency with less trauma and postoperative pain for the dog.

GnRH releasing implants desensitize the pituitary to the stimulatory effects of GnRH and block testicular function (testosterone and sperm production). Driancourt and Briggs provide an explanation and evaluation of the efficacy and safety research for two GnRH agonists: deslorelin and azagly-nafarelin. The deslorelin releasing implant is an adjunct or alternative for surgical sterilization of male dogs formulated for either at least six or 12 months. Azagly-nafarelin is a solid implant (Gonazon^®^) delivered 6-months to 1 year of suppressed fertility; however, it is not commercialized. The authors reviewed data on reversibility and long-term treatment and reported the treatment as having few adverse effects.

## Behavioral and health effects of neutering

Much of the increased interest in spay-neuter stems from evidence of some adverse consequences. Bain addressed relationships among surgical procedures, animal behaviors, stressful animal experiences, and ethical dilemmas. Some surgical procedures can affect an animal's behavior, such as castration, and some pose an ethical dilemma, such as ear cropping and declawing. For these varied procedures, ameliorating pain, decreasing stressful experiences for the animal, and identifying and treating concurrent problem behaviors are hallmarks that can improve the welfare for the animal.

Hart et al.(a) evaluated mixed breed dogs in five body weight categories for their risks of one or more joint disorders associated with age of neutering, as compared with intact dogs. Only in the three weight categories of mixed-breed dogs weighing 20 kg or more, neutering before 1 year generally was significantly associated with risks of one or more joint disorders above that of dogs left intact, commonly to 3 times the level of intact dogs, with sex differences in the degrees of joint disorders associated with neutering.

In an evaluation of patient veterinary records for 35 breeds of dogs and effects of neutering age on joint disorders and cancers, Hart et al.(b) found major breed differences in vulnerability to neutering. In most cases, the caregiver can choose the age of neutering without increasing the risks of these joint disorders or cancers. Subsequently, Hart and Hart reported that an additional five large breeds differed in their vulnerability to joint disorders and cancers with early neutering: German Shorthaired/Wirehaired Pointer; Mastiff, Newfoundland, Rhodesian Ridgeback, and Siberian Husky. Updated guidelines in this article covered 40 dog breeds. These results further emphasize the importance of personalized decisions regarding the neutering of dogs, considering the dog's breed, sex, and context.

Hart and Hart reviewed the ancient practice of castrating male animals and humans. Currently, the findings of breed-specific and sex-specific effects for age of neutering dogs have prompted considering a new paradigm with regard to this practice. This involves dealing with each individual dog in a personalized manner that includes consideration of the lifestyle situation when deciding upon the appropriate age of neutering to avoid increasing the risk of a joint disorder or cancer above that inherent for the breed.

For some inherited conditions, Oberbauer et al. reported that neutering is associated with an increased risk of expression. Neutering has also been associated with altered metabolism and a predisposition for weight gain in dogs, which may confound the detected risk association between neutering and disease expression. This article summarizes the effects of neutering on cancer, orthopedic, and immune disorders in the dog and also explores the potentially exacerbating factor of body weight.

When selecting and training service dogs, Zlotnick et al. found that those dogs neutered at < 7 months had more than twice the risk for health-related dismissals as dogs neutered at any older age and this pattern held for orthopedic dismissals. Labradors were at higher risk for orthopedic-related dismissal than golden retrievers and all other breeds.

Early studies had shown improvements in male aggression after castration (8), but recent studies reveal greater fear aggression to strangers by neutered than intact males (9). Shorter exposure to gonadal hormones has been associated with greater fear and aggression for both males and females (10, 11), and longer exposure to gonadal hormones associated with fewer nuisance behaviors and health problems for both sexes (12). This growing body of research suggests that there is a relationship between dogs' age at neuter and the incidence of health and behavioral problems.

## In summary

Issues regarding spaying and neutering dogs have become increasingly complex as more knowledge arises on interactions with diseases, behavior, welfare, and general health of dogs. The new paradigm involves assessing the lifestyle and entire situation of the particular dog as one decides whether and when to neuter a specific dog.

## Author contributions

LH: Conceptualization, Writing – original draft, Writing – review & editing. BH: Conceptualization, Writing – review & editing. MK: Writing – review & editing. KA: Writing – review & editing.
